# Mr. Harrup, on the Treatment of Burns and Scalds

**Published:** 1807-01

**Authors:** Robert Harrup

**Affiliations:** Chobham


### *To the Editors of the Medical and Physical Journal.*

GENTLEMEN,

PERHAPS the opinions of practitioners have been never more divided on any practical subject than on the treatment of burns and scalds. Many years ago cold applications to the injured parts were recommended, and had many advocates and a few opponents. Lately, Doctor Kentish brought forward a mode of practice, apparently, directly opposite; and which, from the testimony of several, has been attended with success. Dr. Kinglake has now called the attention of the medical world to this disputed point, and the parties may now be fairly said to have joined issue. It is to be hoped that, through the medium of your Journal, a sufficient stock of facts will be collected to set the question at rest; for, as it is justly observed by Dr. Kinglake, "in all controverted points, the ultimate appeal legitimately lies with facts." Many years ago I adopted a mode of treatment, which far exceeded my expectations, and of which a short account is given in the London Medical Magazine and Review, vol. vi. p. 147.

I find nothing more is necessary than to keep the injured parts covered with several folds of old linen well soaked in a weak solution of cerussa acetata. About one drachm of the salt to a pint of cold water is in general sufficient. The addition of the saturnine preparation undoubtedly accelerates the cure by contributing to prevent inflammation and suppuration, whilst the reduced temperature relieves from pain in a much shorter time than any other application. So uniform has the success of this application been, that in a great number of very extensive burns and scalds, not one has occurred, where I had occasion to change the method of treatment. For obvious reasons, in young subjects, the lotion is not applied near the mouth.

Where deep eschars occur I have been sometimes under the necessity of applying poultices, but in every injury of this kind they ought to be used as seldom as possible. The internal remedies I have found necessary to administer are those which obviate febrile symptoms, and sometimes anodynes to allay irritation. From long experience I would recommend this plan of cure as superior in all respects to every other with which I am acquainted. With

 regard regard to the method recommended by Dr. Kentish, I must confess, I have had no experience, excepting in one case, which was attended with circumstances that will effectually deter me from a repetition.

Having heard and read a great deal of the wonderful efficacy of this fiery method of treating burns, I was willing to give it a trial, not from any idea of its superiority over that which I had pursued so long, but merely to satisfy myself whether a mode of treatment, seemingly so opposite, would succeed equally well. The following case soon occurred.

About eighteen months since, a farmer's boy, sixteen years of age, early one morning was searching in a drawer containing a quantity of loose gun-powder, and dropt a lighted candle into it. Before he could withdraw his head the explosion took place. He was brought to me three hours after the accident. The whole of his face, to the ears, with the right hand and wrist, were scorched and black, and a considerable tumefaction had taken place, which closed the eye-lids. He complained of little or no pain. The injured parts were well bathed with warm ol. terebinth. and old linen, dipped in the same liquid, was applied over the whole, except to the eye-lids. He had an anodyne draught at bed-time, and was directed to have the dressings frequently moistened with the oil without removing them. He passed a comfortable night, and when brought to my house next morning, was free from pain. Several large pieces of the discoloured and scorched cuticle came off on the removal of the dressings. Slips of old linen dipped in warm ol. terebinth. in which a small quantity of the ung. resin. flav. had been dissolved, were now applied. He instantly complained of violent pain, which in a very few minutes increased to a higher degree than I ever witnessed, if we may judge from appearances. His screams were dreadful. He threw himself on the floor, and was only by force prevented from injuring himself. In this state he continued upwards of half an hour, and it was some hours before he was tolerably easy. The cure was afterwards completed in fifteen or sixteen days by the application of powdered chalk, and the ung. resin. flav.

What would have been the consequence had this poor patient been a delicate female or an infant?

I am, &c.

ROBERT HARRUP.

